# Architecture of lower leg muscles in children: Reference curves and potential mechanisms of growth

**DOI:** 10.1111/joa.70082

**Published:** 2025-12-01

**Authors:** Brian V. Y. Chow, Suzanne Davies, Catherine Morgan, Caroline D. Rae, David I. Warton, Iona Novak, Ann Lancaster, Gordana C. Popovic, Rodrigo R. N. Rizzo, Claudia Y. Rizzo, Iain K. Ball, Robert D. Herbert, Bart Bolsterlee

**Affiliations:** ^1^ Neuroscience Research Australia (NeuRA) Sydney New South Wales Australia; ^2^ School of Biomedical Sciences University of New South Wales Sydney New South Wales Australia; ^3^ Cerebral Palsy Alliance Research Institute, Discipline of Child and Adolescent Health The University of Sydney Sydney New South Wales Australia; ^4^ School of Psychology University of new South Wales Sydney New South Wales Australia; ^5^ School of Mathematics and Statistics University of New South Wales Sydney New South Wales Australia; ^6^ Evolution & Ecology Research Centre University of New South Wales Sydney New South Wales Australia; ^7^ Faculty of Medicine and Health The University of Sydney Sydney New South Wales Australia; ^8^ Stats Central, Mark Wainwright Analytical Centre University of New South Wales Sydney New South Wales Australia; ^9^ School of Health Sciences University of New South Wales Sydney New South Wales Australia; ^10^ Philips Australia & New Zealand North Ryde New South Wales Australia; ^11^ Graduate School of Biomedical Engineering University of New South Wales Sydney New South Wales Australia

**Keywords:** childhood development, diffusion tensor imaging, growth, muscle architecture

## Abstract

Muscle architecture [i.e. physiological cross‐sectional area (PCSA), fascicle length and pennation angle] changes significantly during childhood. Previous studies have described the architecture of selected muscles in small samples of children over narrow age ranges, but a comprehensive analysis of the distribution of muscle architectural parameters during childhood development is currently lacking. The primary aim of this study was to estimate age‐ and sex‐conditional distributions (reference curves) of architectural parameters of seven lower leg muscles (soleus, medial gastrocnemius, lateral gastrocnemius, tibialis anterior, tibialis posterior, flexor digitorum longus and flexor hallucis longus) in typically developing children aged 5–15 years. We used anatomical and diffusion‐weighted magnetic resonance imaging to quantify the three‐dimensional architecture and aponeurosis dimensions of seven lower leg muscles in 192 typically developing children aged 5–15 years. Quantile regression with b‐splines was used to estimate muscle‐ and sex‐specific reference curves for PCSA, fascicle lengths and pennation angles. In the median 15‐year‐old, PCSAs were 3.0–4.7 times (range is across muscles and sexes), and fascicle lengths were 1.1–1.7 times that of the median 5‐year‐old, respectively. Thus, lower leg muscle volumes (product of PCSA and fascicle length) increase primarily through transverse growth, rather than longitudinal growth, especially in children above 5 years of age. There was considerable overlap in PCSA, fascicle length and pennation angle distributions between boys and girls at all ages. Further analysis showed that longitudinal growth of muscle‐tendon units is achieved primarily by lengthening of intramuscular aponeuroses and that aponeurosis surface areas scale in proportion with PCSA. The reference curves presented here provide normative data for muscle architecture in children and provide insights into the mechanisms of childhood muscle growth.

## Graphical abstract

Muscle architecture (physiological cross‐sectional area, fascicle lengths and pennation angle) was measured from multimodal MRI scans of the lower legs of 197 children aged ≤15 years. We showed that lower leg muscle volumes (product of PCSA and fascicle length) increase primarily through increases in PCSA (transverse growth) rather than increases in fascicle length (longitudinal growth), especially in children above 5 years of age.

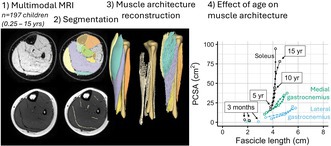

## 1 INTRODUCTION

Skeletal muscles change in size, shape and architecture throughout the lifespan (Blazevich, 2006; Zapaishchykova et al., 2023), but the most dramatic changes occur during childhood (Bell et al., 2022; Chow et al., 2023). Prenatal growth in mammalian skeletal muscles has been largely attributed to the addition of new muscle fibres or hyperplasia (MacCallum, 1898; Montgomery, 1962; Pearson, 1990; Stickland, 1981; White & Esser, 1989). In contrast, postnatal growth is primarily attributable to the serial and parallel addition of new sarcomeres to existing muscle fibres, a process known as muscle fibre hypertrophy (Abernethy et al., 1994; Alnaqeeb & Goldspink, 1987; Rowe & Goldspink, 1969). The addition of sarcomeres in series increases the length of the muscle fibres (longitudinal growth) and extends the range of lengths over which the muscle can generate force (Winters et al., 2011). The addition of sarcomeres in parallel increases muscle fibre cross‐sectional area (transverse growth) and thus the physiological cross‐sectional area (PCSA; the sum of cross‐sectional areas of all fibres in a muscle) of the muscle and its force‐generating capacity (Powell et al., 1984; Siebert et al., 2015). During childhood development, skeletal muscles need to grow both in longitudinal and transverse directions to accommodate longitudinal bone growth and increase in body mass (Benard et al., 2011).

Much of current understanding of growth‐related changes in mammalian skeletal muscle architecture is based on studies of rats and rabbits (Dekoning et al., 1987; Siebert et al., 2017; Swan et al., 2014; Willems & Huijing, 1992; Woittiez et al., 1986). Most of these studies measured fascicle lengths at one or a small number of locations in the muscle belly. An important exception was a study in which high‐resolution measurements of the 3D trajectories of a large number of muscle fascicles were obtained from several leg muscles of rabbits of different ages (Siebert et al., 2017). Surprisingly, Siebert et al. (2017) found that, between 21 and 100 days of age (when skeletal growth in rabbits is almost complete), fascicle lengths of the flexor digitorum longus and the medial and lateral gastrocnemius remained almost unchanged, while fascicle lengths in the tibialis anterior doubled. Moreover, the mean ratio of tibialis anterior tendon and muscle fascicle lengths decreased from 2.0 to 1.5, whereas the mean ratio of flexor digitorum longus tendon and muscle fascicle lengths increased from 8 to 13. This study showed definitively that muscles can undergo major changes in architecture during postnatal growth and that growth‐related changes in architecture differ between muscles. The finding that patterns of growth appear to be muscle‐specific suggests that the specific patterns of growth observed in animal muscles may not directly apply to human muscles.

Over 35 cross‐sectional studies have used imaging techniques to study the architecture of children's skeletal muscles, most frequently the medial gastrocnemius muscle (Bell et al., 2022). Medial gastrocnemius architecture has been measured in children in 2D using ultrasound imaging (Barber et al., 2011; Benard et al., 2011; Herskind et al., 2016; Weide et al., 2015) and in 3D using diffusion tensor imaging (Chow et al., 2023; D'Souza et al., 2019). The data reported in most of these studies appear to support the conclusion that medial gastrocnemius fascicles grow predominantly in cross‐section rather than in length. For example, by comparing measurements obtained from five infants to measurements obtained in adults from a different study, Chow et al. (2023) found a 1.7‐fold difference in fascicle length and a 36‐fold difference in PCSA. A limitation of this and all other studies on children's muscle architecture is that relatively small sample sizes (up to 50 children; Mohagheghi et al., 2008) have been used. Another limitation is that only the medial gastrocnemius muscle has been studied comprehensively. Thus, we lack a complete understanding of the changes in muscle architecture that occur during childhood development.

As part of a large‐scale magnetic resonance imaging (MRI) study on the growth of muscles in the human lower leg (see Herbert et al., 2019 for the protocol), the present study aimed to provide normative data for muscle architecture parameters (PCSA, fascicle length and pennation angle) of lower leg muscles during typical childhood development. To this end, diffusion‐weighted MRI data from the lower legs of 192 typically developing children were analysed to reconstruct the 3D architecture of seven lower leg muscles and measure their PCSA, mean fascicle length and mean pennation angle (Bolsterlee et al., 2019; Damon et al., 2017; Froeling et al., 2012; Zhang et al., 2023). The measurements were used to create *reference curves* for architectural parameters, that is, a description of the age‐ and sex‐conditional parameter distributions. The data set is subsequently used to explore mechanisms of muscle‐tendon growth (secondary aims). At the muscle level, we explore the contribution of longitudinal (i.e. changes in fascicle length) and transverse (i.e. changes in PCSA) growth of fascicles to overall volume growth of muscles. At the muscle‐tendon level, we explore how the morphologies of lower leg muscle‐tendon units adapt to longitudinal tibia growth. Specifically, we determine (1) the contribution of extramuscular tendons, aponeuroses and fascicles to length changes in muscle‐tendon units during longitudinal tibia growth and (2) the relationship between PCSA and aponeurosis areas.

## 2 METHODS

### 2.1 Participants

Participants were infants born at term and aged ≤4 months and typically developing children (specifically, children who did not have a musculoskeletal growth disorder) aged 5–15 years, residing in New South Wales (NSW) or the Australian Capital Territory (ACT) in Australia (Table 1). We report data by sex as assigned at birth (i.e. biological sex) as reported by the parent or guardian. Children were recruited from the community through advertisements and newsletters. The study was approved by the Sydney Children's Hospital Network Human Research Ethics Committee (HC number: 2019/ETH11705) and adhered to the Declaration of Helsinki. Written informed consent was provided for each participant by a parent or guardian.

Muscle volumes obtained from anatomical MRI scans of this cohort have been reported previously (Chow et al., 2023, 2024). Architectural parameters obtained from diffusion‐weighted images, which are the focus of the present study, have not been published previously, with the exception of medial gastrocnemius architecture in infants (Chow et al., 2023).

**TABLE 1:** Participant characteristics.

	Infants		Children	
	Boys	Girls	Boys	Girls
Number of participants (% of total)	3 (60%)	2 (40%)	111 (58%)	81 (42%)
Age (years)	0.2 ± 0.1 (0.1–0.3)	0.2 ± 0.0 (0.2–0.3)	10.3 ± 2.5 (5.4–14.9)	10.4 ± 2.7 (5.1–15.0)
Tibia length (cm)	10.2 ± 0.6 (9.6–10.9)	9.3 ± 0.2 (9.2–9.5)	32.8 ± 4.7 (22.5–43.0)	32.4 ± 4.4 (23.7–41.5)
Body height (cm)	59.1 ± 3.0 (57.0–62.5)	56.6 ± 3.4 (54.2–59.0)	144.2 ± 16.5 (108.9–181.5)	143.5 ± 16.5 (108.4–179.4)
Body weight (kg)	6.8 ± 1.0 (6.0–7.9)	4.8 ± 0.8 (4.2–5.3)	37.1 ± 11.7 (17.0–76.4)	37.8 ± 12.8 (17.3–73.1)

*Note*: Values are mean ± standard deviation (minimum–maximum).

### 2.2 Image acquisition

Anatomical MRI scans (T1‐weighted and mDixon) and diffusion‐weighted imaging (DWI) scans were obtained from one randomly selected lower leg in a 3T Philips Ingenia CX MRI scanner (Philips Healthcare, Best, The Netherlands) using a dS torso/anterior coil and dS posterior coil with 28 channels (see Table 2 for scan settings). Children above 5 years of age lay supine with a foam pillow under the knee. The pillow prevented compression of the calf muscles by the scanner bed and positioned the knee in 30° flexion. The foot was strapped to a footplate that held the ankle in 15° plantarflexion (i.e. 15° more plantarflexed than with the sole of the foot perpendicular to the tibial shaft). The position of the knee and ankle was verified with a handheld goniometer. Infants were scanned using the feed and wrap technique (see Chow et al., 2023 for details) with the knee in a nearly extended position and the ankle in a relaxed position.

**TABLE 2:** Magnetic resonance imaging parameters.

	mDixon	T1‐weighted	Diffusion‐weighted
Sequence	2‐point 3D T1‐fast field Echo	2D turbo spin‐echo (TSE)	Echo‐planar imaging (EPI)
Repetition time (ms)	6.0–6.2	629.1–737.6	3210–13,000
Echo time (ms)	3.5/4.6	12	45
Number of signal averages	2	2	2–3
Acquisition voxel size (mm)	1 × 1 × 2	0.7 × 0.85 × 5	2.5 × 2.5 × 5
Acquisition matrix	160 × 160	228 × 192	64 × 62
Reconstructed voxel size (mm)	1 × 1 × 1	0.4 × 0.4 × 5	1.67 × 1.67 × 5
Number of slices	290–485	58–97	48–90
Scan duration (s)	164–276	133–245	164–1001
Gradient scheme	–	–	12 directions on a hemisphere
*b*‐value (s/mm^2^)	–	–	500 (b0 with 0)
Diffusion gradient time (Δ/δ, ms)	–	–	21.8/12.4

T1‐weighted and mDixon MRI scans, used for anatomical reference and segmentation of aponeurosis, muscles and bones, were acquired in the transverse plane, covering the volume from the femoral condyle (just proximal to the origins of the gastrocnemii) to the calcaneus (just distal to the Achilles tendon insertion). DWI scans were acquired at slightly reduced volumes, covering the length of the tibia but typically excluding the ankle and knee joints. Image acquisition, including positioning children in the scanner, typically took 30–40 min.

### 2.3 Image analysis

DWI scans were preprocessed using MRtrix version 3.0.4 (50). DWI scans were first denoised using Marchenko‐Pastur principal component analysis (Veraart et al., 2016), followed by corrections for eddy current and motion distortions (Andersson & Sotiropoulos, 2016) and echo‐planar imaging (EPI) distortions (Andersson et al., 2003). The DWI scan was then upsampled with linear interpolation to the resolution of the mDixon scan (1 × 1 × 1 mm).

Small misalignments between T1‐weighted, mDixon and DWI scans caused by participant movement between scans were corrected by rigidly registering the mDixon water image to the b0 image of the preprocessed DWI scan and by rigidly registering the T1‐weighted scan to the registered mDixon scan (Greve & Fischl, 2009). The accuracy of the alignment was visually verified in image software ITK‐SNAP (Yushkevich et al., 2006).

**FIGURE 1:**
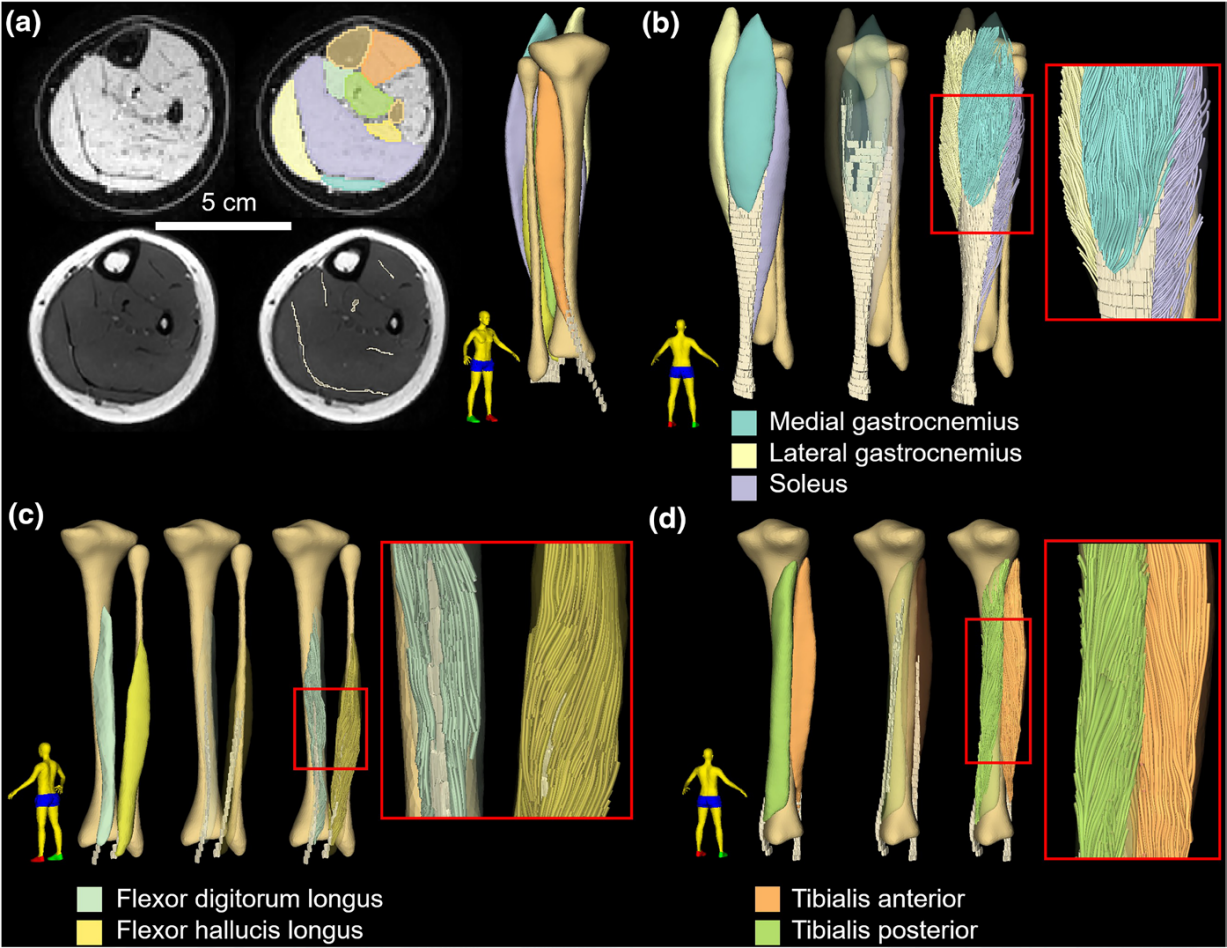
Muscle architecture reconstructions from multi‐modal MRI data. (a) Transverse slice of an mDixon water image (top) and T1‐weighted image (bottom) obtained approximately midway between the ankle and the knee of the right leg of a 5‐year‐old boy. The images on the right show the original image with segmentations of muscles (top) and aponeuroses (bottom) as overlays. Three‐dimensional reconstructions of the surface of the tibia, muscles and aponeuroses are shown on the right. (b–d) Examples of muscle architecture reconstructions of the triceps surae (b), flexor digitorum longus and flexor hallucis longus (c) and the tibialis anterior and tibialis posterior (d). The panels show, from left to right, the muscle surfaces and aponeuroses, the semi‐transparent muscle surfaces and aponeuroses (highlighting the intramuscular aponeuroses used to constrain tractography; see main text), the whole‐muscle fascicle reconstructions, and an enlarged view on part of the fascicle reconstructions (red rectangle). The tibia and fibula are displayed for reference purposes (fibula not shown in d). The human figure in the bottom left shows the view angle (the right foot is green).

The tibia, fibula and seven muscles (soleus, medial gastrocnemius, lateral gastrocnemius, tibialis anterior, tibialis posterior, flexor digitorum longus and flexor hallucis longus) were segmented automatically from mDixon images (Figure 1a) using a previously trained nnU‐Net (Isensee et al., 2021). Details are provided elsewhere (Chow et al., 2024; Zhu et al., 2021). A separate nnU‐Net was trained on T1‐weighted images to segment tendons and aponeuroses (Figure 1a). T1‐weighted images were used to segment aponeuroses because the T1‐weighted scans had a higher transverse‐plane resolution than the mDixon images and aponeuroses are thin, longitudinally oriented structures (Bird et al., 2019). All segmentations were visually inspected and corrected where necessary.

### 2.4 Muscle architecture measurements

The trajectories of muscle fascicles were reconstructed in 3D from DWI scans using the anatomically constrained tractography framework built into MRtrix (Smith et al., 2012), which we recently adapted for muscle architecture measurements (Zhang et al., 2023, 2024). For muscles with aponeuroses that lie on the surface of the muscle (medial and lateral gastrocnemius), the framework ensures that fascicle reconstructions have attachments on the muscle surface (Figure 1). For muscles with aponeuroses that run centrally through the muscle belly (tibialis posterior, tibialis anterior, flexor hallucis longus and flexor digitorum longus and the soleus), the framework ensures that fascicle reconstructions originate from intramuscular aponeuroses. For each muscle, we tracked 5000 fascicles that satisfied anatomical constraints, using the following tractography settings: step size 1 mm, maximum turning angle 10°, fractional anisotropy >0.1, 10 ≤ length ≤ 250 mm. Note that the fascicle reconstructions from tractography, referred to in the remainder of this paper as ‘fascicles’, do not have a direct correspondence to ‘anatomical’ fascicles but can nonetheless be used to infer the lengths and orientation of anatomical fascicles (Bilston et al., 2019).

As found previously (Oudeman et al., 2016), some fascicles in muscles with internal aponeuroses (e.g. tibialis anterior) displayed unrealistic trajectories that tracked the aponeuroses for a significant portion of their length. We identified these fascicles using a trajectory constraint which ensured that fascicles did not depart too far from a linear trajectory between aponeuroses. Specifically, for all points on a fascicle, the distance along the fascicle and the distance to the nearest aponeurosis were calculated. A quadratic curve was then fitted to the relationship between the distance along the fascicle and the squared distance to the aponeurosis. We determined empirically that fitted curves with an *R* ^2^ ≥ 0.7 followed plausible trajectories, so fascicles with *R* ^2^ < 0.7 were excluded from further analyses.

The length of each fascicle was calculated as the sum of the length of all fascicle tract segments. The pennation angle of a fascicle was calculated as the angle in 3D between the straight line connecting the end points of that fascicle and the muscle's line of action. For the tibialis anterior, tibialis posterior, flexor hallucis longus and flexor digitorum longus, the line of action was defined as the direction of the first principal component of the centroid of all voxels of the intramuscular portion of the aponeurosis. For the medial gastrocnemius, lateral gastrocnemius and soleus, the line of action was defined as the direction of the first principal component of the centroid of all voxels inside the muscle. A muscle's fascicle length and pennation angle were defined as the mean fascicle length and mean pennation angle of all fascicles in that muscle, respectively. Mean fascicle length and pennation angle measurements were previously shown to have good between‐day reliability, with a mean intraclass correlation coefficient across muscles of 0.81 for fascicle lengths and 0.73 for pennation angles (Bolsterlee et al., 2019). Muscle volume was calculated from the segmentation as the product of the volume of a voxel and the number of voxels within that muscle. A muscle's PCSA was calculated as muscle volume divided by muscle fascicle length.

Ideally, we would have measured muscle architecture in infants at the same ankle and knee position as in older children, but we did not control joint angles in infants. Our previous attempt (Chow et al., 2023) to correct muscle architecture measurements for joint position in the infant medial gastrocnemius resulted in small differences in fascicle lengths (<3 mm) and PCSA (<0.1 cm^2^). Because the effect of correction is likely to be small, and because we could not measure the moment arms required for the correction of muscles other than the triceps surae muscles, we did not correct infant muscle architecture measurements here.

### 2.5 Contribution of muscle‐tendon unit components to longitudinal bone growth

To investigate the extent to which fascicles, aponeuroses and extramuscular tendons adapt to changes in tibia length during growth, we measured tibia length and, for all seven muscles, tendon, aponeurosis and fascicle lengths, projected along the tibia's longitudinal axis. Tibia length was calculated as the length of the bone's long axis, determined using principal component analysis on the vertices of a 3D surface model of the tibia (Chow et al., 2024). Tendinous insertions on the foot were usually outside the field of view of the scan, so we measured the component of tendon length along the tibia as the distance from the most distal point of the muscle to the most distal point of the tibia. The longitudinal component of fascicle length was calculated as the product of the muscle's mean fascicle length and the cosine of the pennation angle. For muscles with an internal aponeurosis (tibialis anterior, tibialis posterior, flexor hallucis longus and flexor digitorum longus), which can reliably be segmented from T1‐weighted scans (Bird et al., 2019), aponeurosis length was calculated as the distance between the most distal point of the muscle (i.e. the muscle‐tendon junction) and the most proximal point of the aponeurosis. For the medial gastrocnemius, lateral gastrocnemius and soleus, aponeurosis length was estimated by subtracting the longitudinal component of fascicle length from the muscle belly length (distance between the most proximal and most distal points of the muscle belly).

### 2.6 Relationship between PCSA and aponeurosis surface area

The ends of muscle fascicles attach to aponeuroses, so when muscle fascicles grow transversely, aponeurosis dimensions must change too. To investigate the relationship between aponeurosis dimensions and transverse growth of muscle fascicles, we determined the ratio of aponeurosis surface area and PCSA. Aponeurosis surface area was measured as the surface area of a 3D triangulated surface model that enveloped all centroids of the aponeuroses' voxels. This analysis was conducted on the four muscles with internal aponeuroses whose aponeurosis surface area could be measured reliably using previously described methods (Bird et al., 2019). Aponeurosis dimensions were difficult to measure from infant scans because of their small size, so we excluded infant data from analyses that relied on aponeurosis dimensions.

### 2.7 Statistical analysis

Statistical analyses were conducted using R (version 4.3.2) and Rstudio (version 2023.12.0). The relationship between age and muscle architecture (fascicle length, PCSA and pennation angle) was estimated with non‐parametric quantile regression (Muggeo et al., 2013), yielding reference curves describing the distributions (summarised using centile curves) of architectural parameters from age 0–15 years. To capture potential non‐linear relationships between age and muscle architecture, centile curves were modelled with a penalized b‐spline as a basis with the penalty (i.e. the smoothing factor) determined through cross‐validation (Muggeo et al., 2021). For fascicle length and PCSA (but not pennation angle), centile curves were constrained to increase monotonically with age. Reference curves were estimated for the 10th, 50th and 90th centiles, independently for each muscle, sex and architectural parameter. Simultaneous quantile regression ensured that centile curves did not cross. Architectural parameters were log‐transformed prior to fitting, and centile estimates were back‐transformed. The same methods were used to create reference curves for body height, body weight and tibia length. For descriptive purposes, the effect of age on architecture is described by comparing median values estimated at 5 and 15 years (i.e. the ends of the age range studied here). We also provide values for median boys and girls at these ages, but we did not conduct formal statistical tests of the observed differences.

Tibia length, tendon length, aponeurosis length and the longitudinal component of fascicle length were linearly regressed against age for all muscles. Similarly, the ratio of aponeurosis surface area and PCSA was regressed against age for the tibialis anterior, tibialis posterior, flexor hallucis longus and flexor digitorum longus. There were no discernible differences between boys and girls in the effect of age on the lengths of muscle‐tendon units, aponeuroses or fascicles or the ratios of aponeurosis surface areas and PCSA, so data from boys and girls were pooled for these analyses.

## 3 RESULTS

### 3.1 Participants

**FIGURE 2:**
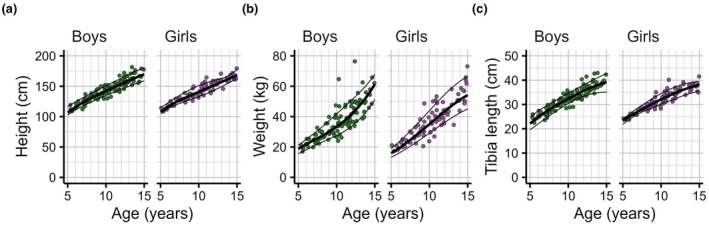
The relationship between age and (a) body height, (b) body weight and (c) tibia length. Each panel shows the 10th (bottom line), 50th (thick line in the middle) and 90th centile curves (top line) and the observations on muscles of individual boys (green, *n* = 114, left panel) and girls (purple, *n* = 83, right panel) on which the curves were fitted.

A total of five infants aged 1.7–3.6 months and 192 children—111 boys and 81 girls aged 5.1–15.0 years—participated in this study (Table 1, Figure 2). In children above 5 years of age, body height ranged from 108 to 182 cm, body weight from 17 to 76 kg and tibia length from 22 cm to 43 cm (Figure 2). Between the median 5‐year‐old and 15‐year‐old boy and girl, respectively, there was a 1.6‐fold and 1.6‐fold difference in body height, a 3.3‐fold and a 3.4‐fold difference in body weight and a 1.8‐fold and a 1.6‐fold difference in tibia length.

### 3.2 Reference curves for muscle architecture

Muscle architecture was successfully measured for all muscles, yielding measurements of fascicle length, PCSA and pennation angle for a total of 1379 muscles (seven muscles for each of five infants and 192 children). Of the 5000 fascicles per muscle that satisfied anatomical constraints, on average 73% (range between muscles: 60% to 92%) satisfied the trajectory constraint and were thus used to calculate a muscle's PCSA, fascicle length and pennation angle.

**FIGURE 3:**
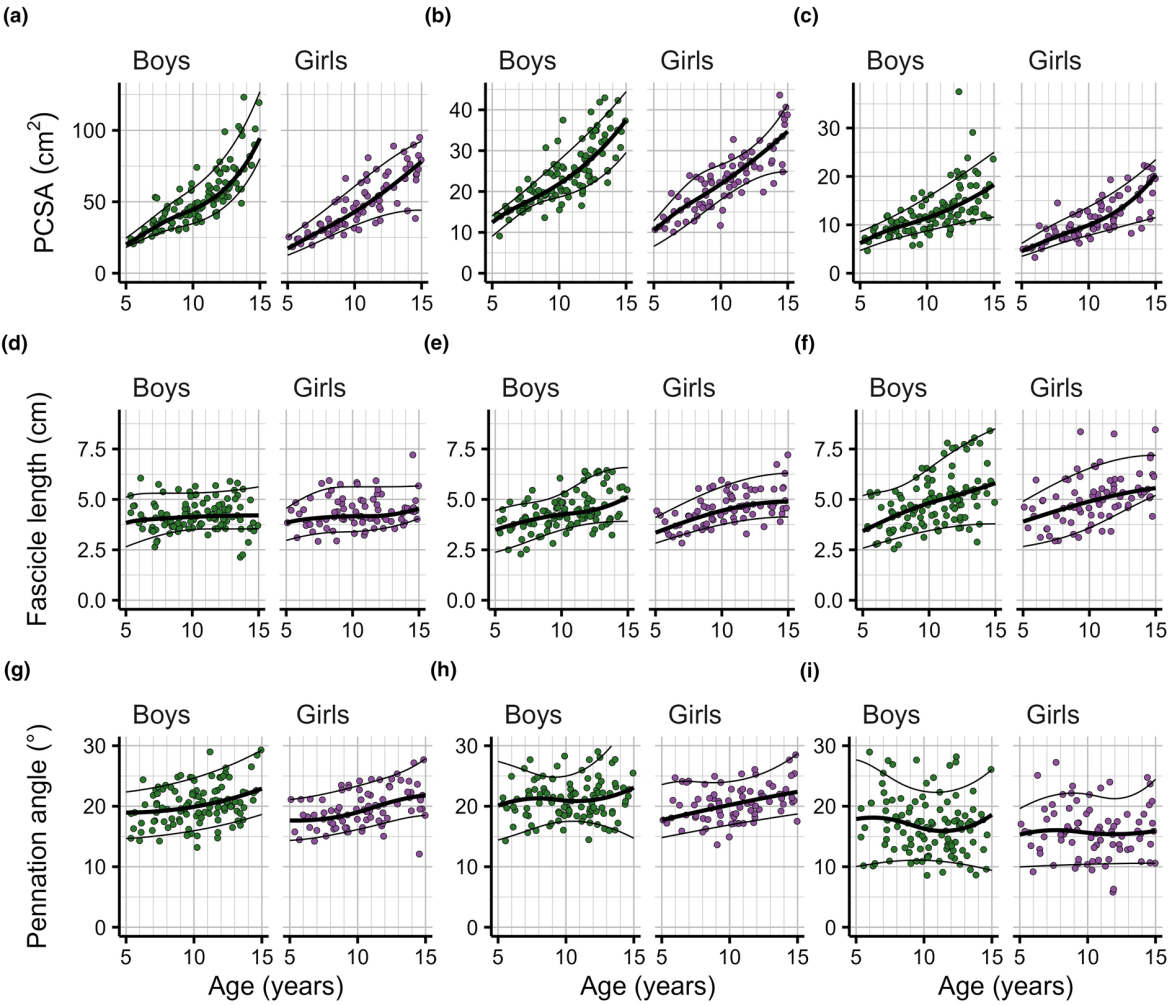
Reference curves for the soleus (a, d, g), medial gastrocnemius (b, e, h) and lateral gastrocnemius muscles, for physiological cross‐sectional area (PCSA, a–c), fascicle length (d–f) and pennation angle (g–i), as a function of age. Each panel shows the 10th (bottom line), 50th (thick line in the middle) and 90th centile curves (top line) and the observations on muscles of individual boys (green, *n* = 114, left panel) and girls (purple, *n* = 83, right panel) on which the curves were fitted.

**FIGURE 4:**
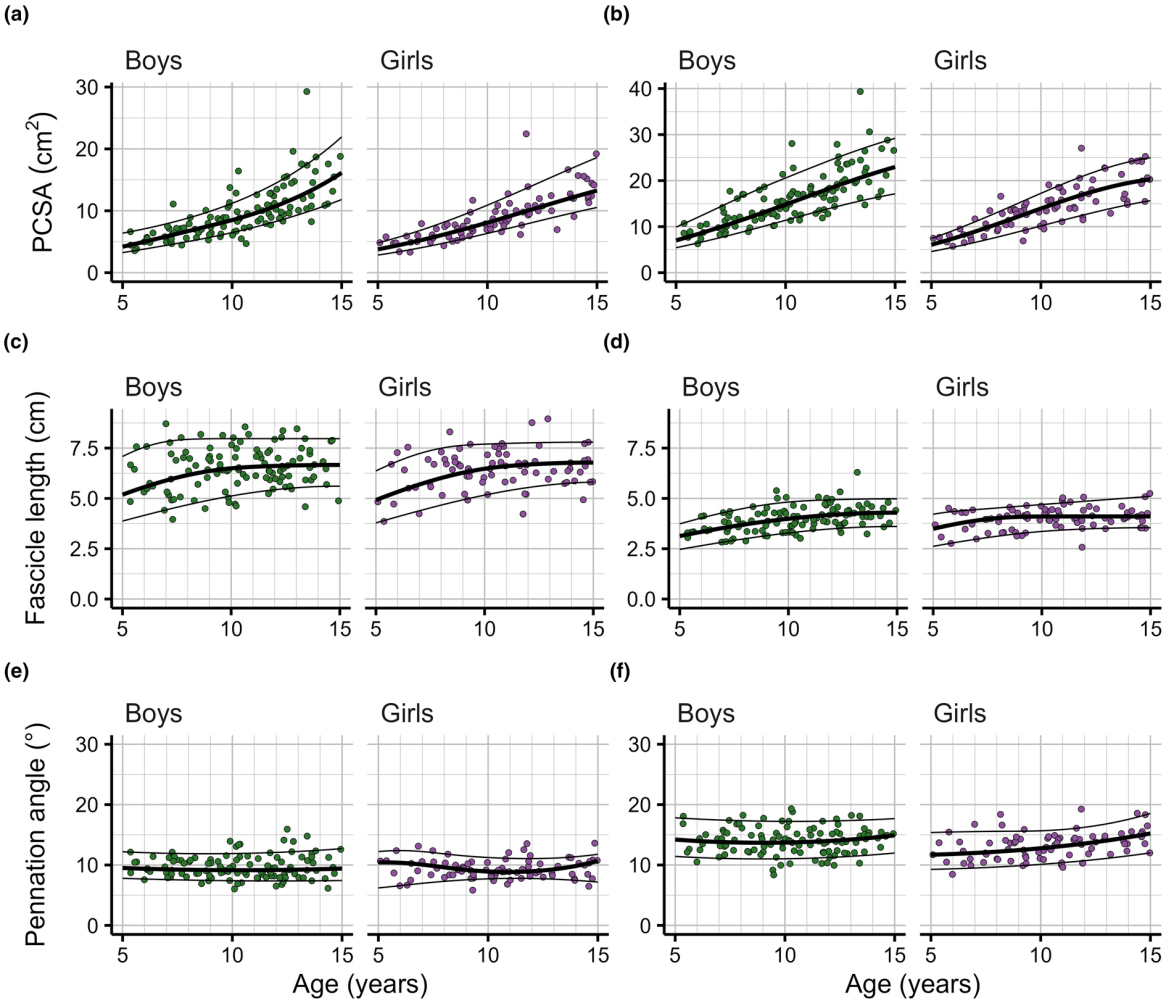
Reference curves for the tibialis anterior (a, c, e) and tibialis posterior muscles (b, d, f), for physiological cross‐sectional area (PCSA, a, b), fascicle length (c, d) and pennation angle (e, f), as a function of age. Each panel shows the 10th (bottom line), 50th (thick line in the middle) and 90th centile curves (top line) and the observations on muscles of individual boys (green, *n* = 114, left panel) and girls (purple, *n* = 83, right panel) on which the curves were fitted.

**FIGURE 5:**
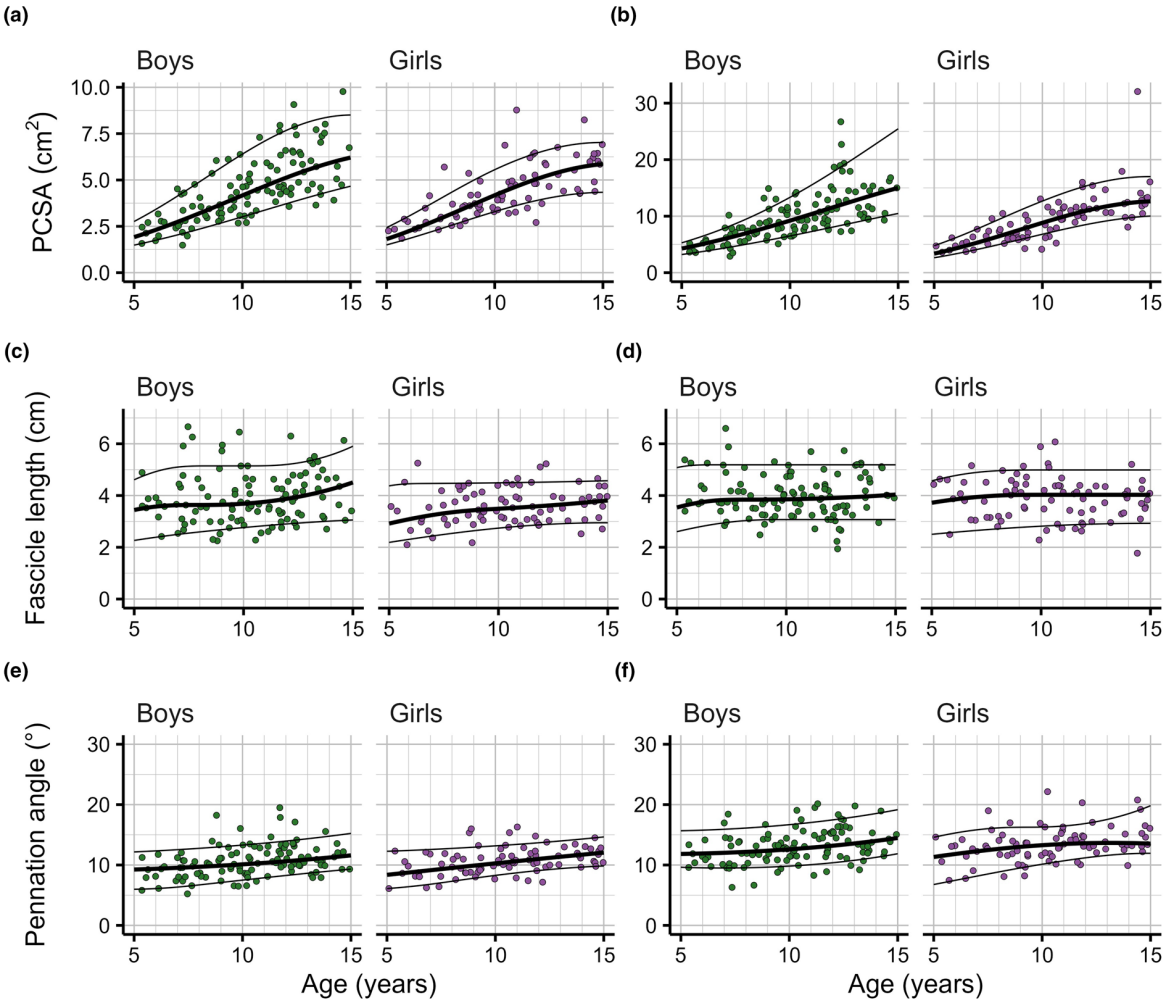
Reference curves for the flexor digitorum longus (a, c, e) and flexor hallucis longus muscles (b, d, f), for physiological cross‐sectional area (PCSA, a, b), fascicle length (c, d) and pennation angle (e, f), as a function of age. Each panel shows the 10th (bottom line), 50th (thick line in the middle) and 90th centile curves (top line) and the observations on muscles of individual boys (green, *n* = 114, left panel) and girls (purple, *n* = 83, right panel) on which the curves were fitted.

Reference curves for PCSA showed a substantial shift of the distribution towards larger values in older boys and girls (Figures 3a–c, 4a,b, 5a,b; see Supplementary Material 1 for larger versions). The PCSAs of the median 15‐year‐old boy and girl were, on average across muscles, 3.5 and 3.7 times larger than in the median 5‐year‐old boy and girl, respectively. This ratio ranged across muscles from 3.0 (lateral gastrocnemius in boys) to 4.7 (soleus in boys). The shape of the reference curves for PCSA was similar in boys and girls, but median values were larger in boys in most muscles and at most ages. On average across muscles, PCSAs in the median boy were greater than in the median girl by a factor of 1.18 (ranging from 1.06 for the flexor digitorum longus to 1.36 for the lateral gastrocnemius) at age 5 and by a factor of 1.11 (ranging from 0.90 for the lateral gastrocnemius to 1.22 for the tibialis anterior) at age 15. Distributions of PCSAs at any given age were wide (e.g. the difference between the 90th and 10th centile values was typically 30%–80% of the 50th centile value), so there was considerable overlap between PCSA distributions of boys and girls at all ages.

Reference curves for fascicle lengths showed that 5‐year‐olds typically have 1.3 to 2.2 (range across muscles and sexes) times longer fascicles than 3‐month‐old infants (Figures 3d–f, 4c,d, 5c,d). Above 5 years of age, fascicle length distributions continued to shift to larger values, but the effect of age was generally small, and differed between muscles. In boys and girls, the fascicle lengths of the median 15‐year‐old were, on average across muscles, 1.3 times greater than in the median 5‐year‐old. This ratio was greatest for the medial gastrocnemius (boys: 1.5, girls: 1.5) and lateral gastrocnemius (boys: 1.7, girls: 1.4) and smallest for the flexor hallucis longus (boys: 1.1, girls: 1.1) and soleus (boys: 1.1, girls: 1.2). Reference curves for fascicle lengths were broadly similar in boys and girls. The difference between the 90th and 10th centile values was 30%–80% of the 50th centile value.

Reference curves for pennation angles showed that for most muscles, pennation angles were larger in 15‐year‐olds than in 5‐year‐olds, by up to 4° for the medial gastrocnemius and 5° for the soleus. However, the effect of age on pennation angle was small compared to the distribution of pennation angles at any given age. The difference between the 90th and 10th centile was typically between 5° and 15° across ages and muscles.

**FIGURE 6:**
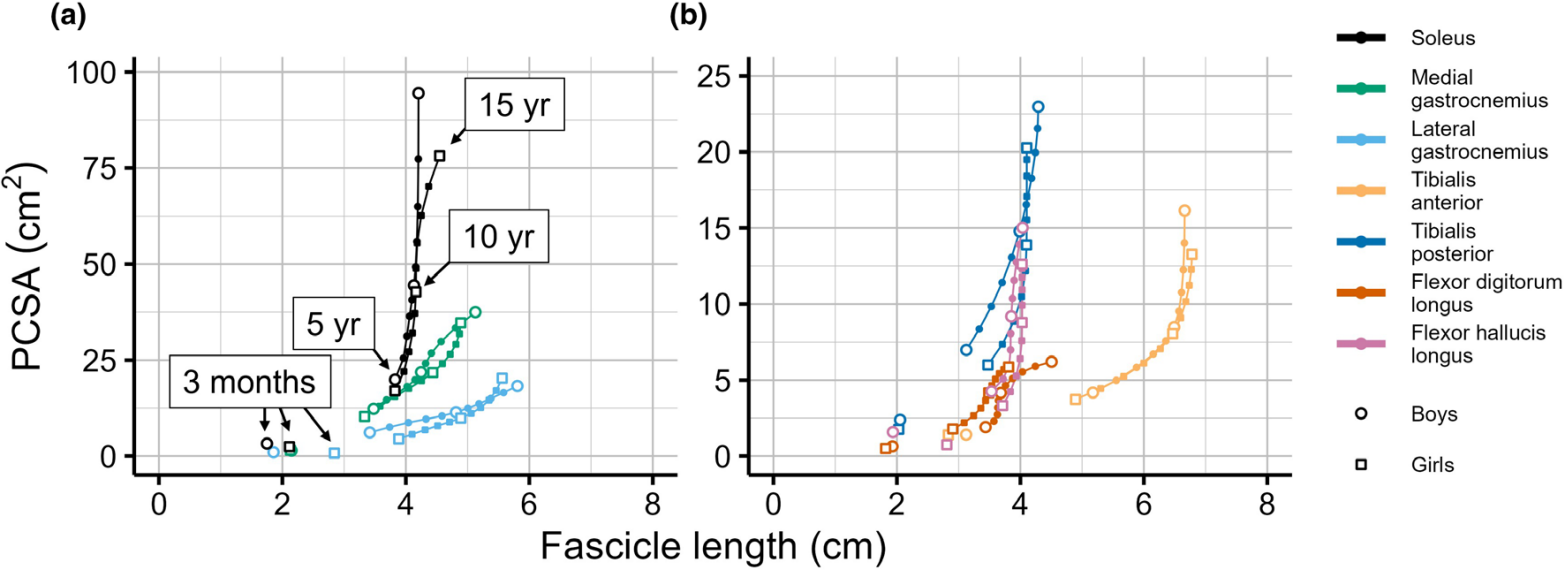
The relationship between physiological cross‐sectional area (PCSA) and fascicle length for (a) the soleus, medial gastrocnemius and lateral gastrocnemius muscles and (b) the tibialis anterior, tibialis posterior, flexor digitorum longus and flexor hallucis longus muscles. The lines connect 50th centile values estimated from age 5 to 15 for 114 boys (circles) and 83 girls (squares). Estimated median values for 3‐month‐old infants (mean age of five infants who participated in this study) are shown as individual data points. Values estimated for whole years are indicated by small circles/squares with larger white circles/squares indicating values obtained at 5, 10 and 15 years.

Muscle volume is the product of mean fascicle length and PCSA. So increases in muscle volume could be mediated either by increases in mean fascicle length (longitudinal growth) or increases in PCSA (transverse growth). Plotting PCSA and fascicle lengths against each other shows that age‐related increases in muscle volume are primarily mediated by increases in PCSA rather than fascicle length for all muscles and at all ages (Figure 6). However, the relative contribution of transverse and longitudinal growth differs between muscles and across ages. For all muscles, the largest increase in fascicle length occurs under 5 years of age. At older ages, and especially above ~10 years of age, changes in PCSA are the primary mechanisms by which muscles grow.

### 3.3 Contribution of muscle‐tendon unit components to longitudinal growth

**FIGURE 7:**
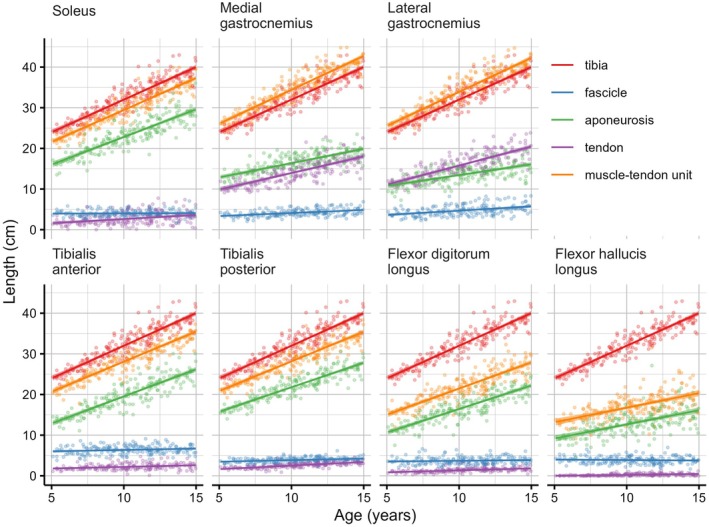
The relationship between lengths of muscle‐tendon unit components and age. Each panel shows for one lower leg muscle the lengths of the longitudinal component of the fascicles (blue), the aponeurosis (green), the extramuscular tendon (purple) and the muscle‐tendon unit (orange; sum of fascicle, aponeurosis and tendon). The length of the tibia (red; same in each panel) is displayed for reference purposes. Note that tendon length excludes the part of the extramuscular tendon distal to the tibia. The solid line is a linear fit of the length against age. The (very narrow) 95% confidence intervals are indicated by the shaded areas.

Above 5 years of age, the lengths of the longitudinal components of the tibia, tendons, aponeuroses and fascicles were linearly, or nearly linearly, associated with age (Figure 7, see Supplementary Material 2 for a table with linear mixed modelling results). Mean tibia length increased by 1.6 cm/year (95% CI: 1.5 to 1.7, *p* < 0.05). For each muscle, the muscle‐tendon unit length (i.e. the sum of the lengths of the longitudinal components of the aponeurosis, tendon and fascicles) remained a constant proportion of tibia length. Fascicles contributed little to the increase in length of the muscle‐tendon unit (maximum of 0.2 cm/year for the lateral gastrocnemius). In the medial gastrocnemius, tendon (0.8 cm/year) and aponeurosis lengthening (0.7 cm/year) contributed approximately equally to overall length changes of the muscle‐tendon unit, whereas in the lateral gastrocnemius, the tendon contributed substantially more (0.9 cm/year) than the aponeurosis (0.5 cm/year). In all other muscles, aponeurosis lengthening accounted for almost all of the age‐related increase in muscle‐tendon unit length.

### 3.4 Relationship between PCSA and aponeurosis area

**FIGURE 8:**
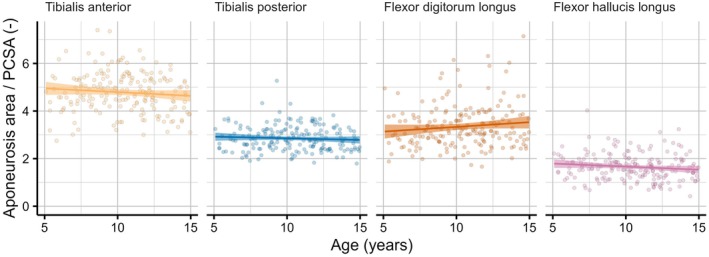
Ratio of aponeurosis surface area and PCSA for four lower leg muscles with internal aponeuroses. The solid line is a linear fit of the ratio versus age. 95% confidence intervals are indicated by the shaded areas.

While the ratio of aponeurosis surface area and PCSA differed substantially between muscles (from 4.8 for the tibialis anterior to 1.7 for the flexor hallucis longus), there was no evidence that this ratio changed with age (Figure 8). That is, the ratio of aponeurosis surface area and PCSA was nearly constant.

## 4 DISCUSSION

To our knowledge, this study is the first to have measured the architecture of multiple skeletal muscles from a large cohort of children over a wide age range. The data were used to construct reference curves which provide normative values for architectural parameters during typical childhood development. The data also provide insights into mechanisms of muscle growth. This Discussion focuses on mechanisms of growth.

A consistent finding across muscles was that age‐related increases in PCSA (3.0 to 4.7‐fold increases from 5 to 15 years) were several times greater than increases in fascicle length (1.1 to 1.7‐fold). Thus, the data provide strong evidence that lower leg muscles change their volume (the product of PCSA and mean fascicle length) during childhood primarily through transverse growth, especially in children above 5 years of age. It has previously been reported that the human medial gastrocnemius experiences primarily transverse growth rather than longitudinal growth in childhood (D'Souza et al., 2019; Weide et al., 2015). The present study shows this is a general feature of lower leg muscle growth in children. In fact, the medial and lateral gastrocnemius were the muscles whose fascicle lengths increased the most above 5 years of age, so the relative contribution of transverse growth was even higher for muscles other than the medial and lateral gastrocnemius.

The dominant role of transverse fascicle growth, presumably through parallel addition of sarcomeres, suggests that muscles primarily adapt to increase their maximum force‐generating capacity, which is generally considered to be proportional to PCSA (Binder‐Markey et al., 2023; Powell et al., 1984). The 3.5‐ and 3.7‐fold difference in PCSA (average across muscles) between the median 5‐ and 15‐year‐old boy and girl, respectively, was close to the 4.4‐fold and 4.0‐fold change in body weight, suggesting that lower leg muscle strength remains an approximately constant proportion of body weight during childhood. Transverse growth of fascicles must be accompanied by changes in the surface areas of aponeuroses to which they attach. Indeed, the ratio of aponeurosis surface area and PCSA remained constant with age, as was previously found in rabbit leg muscles (Papenkort et al., 2020; Siebert et al., 2017). This finding suggests that the stress applied by the muscle to the surface of the aponeurosis at a given contraction level is similar in younger and older children.

The relatively small changes in fascicle length might indicate that muscles can produce force over a smaller range of muscle lengths (as a proportion of tibia length) in older compared to younger children. The 1.3‐fold difference in fascicle length between the median 5‐ and 15‐year‐old was substantially smaller than the 1.6‐ to 1.8‐fold difference in tibia lengths. Thus, if the muscle operating range is proportional to muscle fascicle length (Winters et al., 2011) and lower leg moment arms scale proportionally with tibia length (Alexander et al., 2019), it might be expected that active and passive ankle ranges of motion would be substantially smaller in older children and adults than in young children. While there is some evidence for a minor reduction in passive ankle range of motion with age in children (D'Souza et al., 2019), active range of motion appears to be unaffected by age (Grimston et al., 1993). Inferences about function from muscle architectural data depend on the assumption that the effect of age on fascicle length reflects a serial addition of sarcomeres and the effect of age on PCSA reflects a parallel addition of sarcomeres. However, we did not measure sarcomere lengths, so we do not know if the architectural parameters of older children were measured at the same sarcomere lengths as in younger children. Thus, we cannot say with certainty that fascicle length is proportional to the number of sarcomeres in series or that PCSA is proportional to the number of sarcomeres in parallel. Moreover, to the extent that muscle fascicles transmit force in shear, the functional consequences of fascicle length and PCSA adaptations on whole‐muscle force production are potentially difficult to predict. It may be necessary to use 3D continuum models to accurately predict the functional consequences of age‐related changes in muscle architecture (Rohrle et al., 2012; Wakeling et al., 2020).

If fascicles do not substantially grow in length, how do muscle‐tendon units lengthen to match longitudinal tibia growth? Our data show that, in the lower leg, age‐related increases in the length of those muscle‐tendon units with long tendons are primarily achieved by lengthening of tendon and aponeurosis. In the medial gastrocnemius, tendon and aponeurosis lengthen a similar amount. In the lateral gastrocnemius, there is a slightly larger contribution of tendon growth. In other muscles, age‐related increases in the length of the muscle‐tendon unit are due almost entirely to increases in the length of the intramuscular aponeuroses.

**FIGURE 9:**
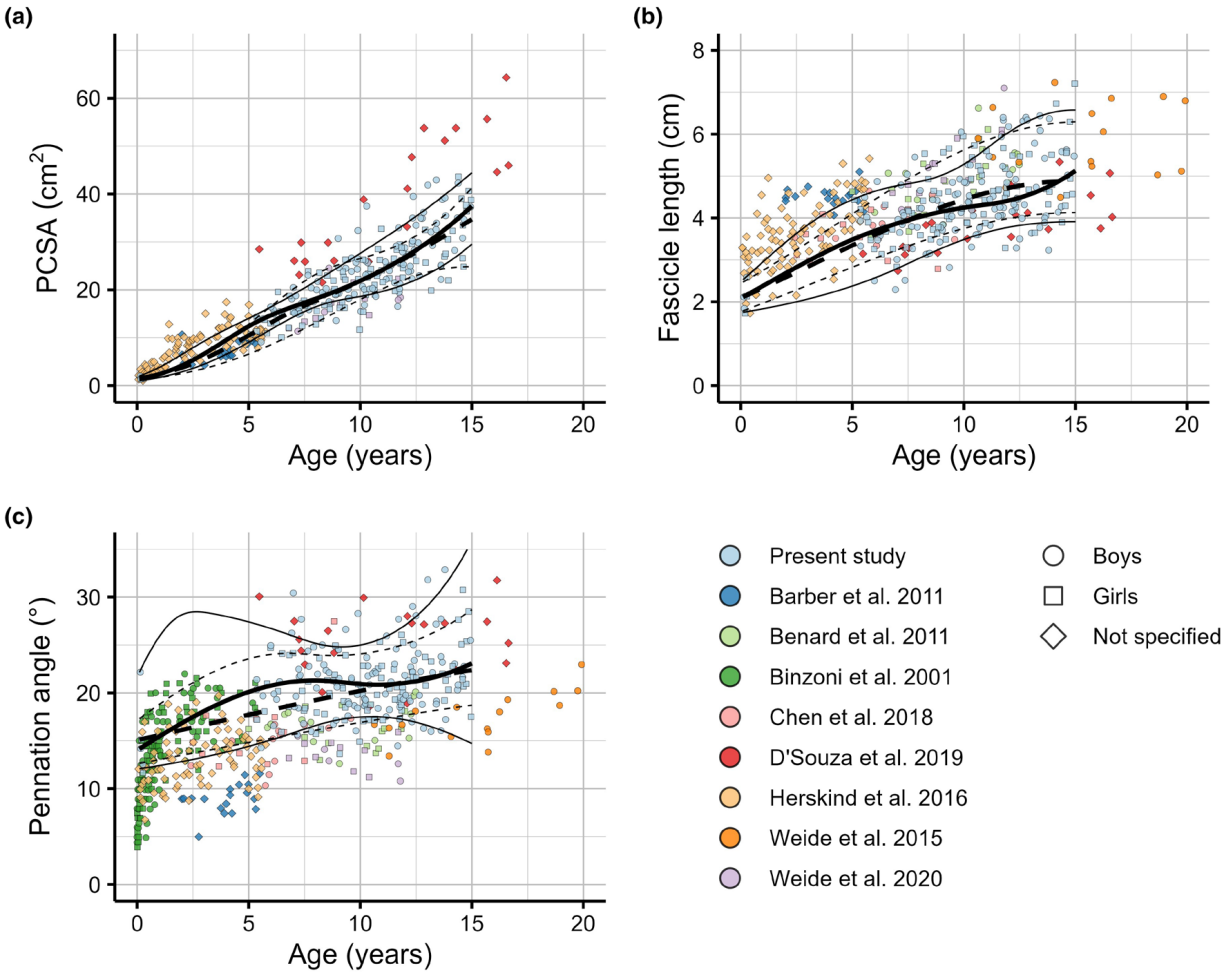
Measurements of medial gastrocnemius architecture reported in nine studies. Data are measurements of (a) physiological cross‐sectional area (PCSA; *n* = 335), (b) fascicle length (*n* = 408) and (c) pennation angle (*n* = 547) obtained from the medial gastrocnemius (MG) muscle in the present study and eight other studies. Data points from other studies were digitised using WebPlotDigitizer v4.5 (Rohatgi, 2021). Five studies (including the present study) measured PCSA. Of those, Herskind et al. (2016) and Barber et al. (2011) used a different definition for PCSA to that used here. For those two studies, we recalculated PCSA by dividing muscle volume by mean fascicle length. The black lines indicate the 50th centile (thick line) and 10th and 90th centile (thin lines) for boys (solid) and girls (dashed) obtained using only data from the present study (same curves as in Figure 3b,e,h).

Comparison of our data with previous studies is only possible for the medial gastrocnemius, as previous studies on muscle architecture during childhood growth have focused almost exclusively on this muscle. Differences in joint positions at which measurements were obtained, study populations and measurement methodology (3D MRI versus 2D ultrasound) make rigorous comparisons between studies difficult. For example, 2D ultrasound provides unbiased but imprecise measurements of 3D fascicle lengths but systematically overestimates pennation angles in the medial gastrocnemius (Bolsterlee et al., 2015) and there is currently no consensus on the location within a muscle from which ultrasound images should be obtained for reliable architecture measurements. Nevertheless, there is reasonable correspondence between our data and data from previous studies (Figure 9; Barber et al., 2011; Benard et al., 2011; Binzoni et al., 2001; Chen et al., 2018; D'Souza et al., 2019; Herskind et al., 2016; Weide et al., 2015, 2020). PCSAs and fascicle lengths measured by Herskind et al. (2016) from 100 typically developing children aged 1 month to 5.8 years were often greater than the 90th centile curves fitted on our data, and pennation angles were often smaller than the 10th centile (Figure 9). We did not have data for children between 4 months and 5 years of age, so the reference curves presented here may underestimate fascicle lengths and PCSAs and overestimate pennation angles for this age range. Alternatively, differences in methodology (they used a combination of 2D and 3D ultrasound) may explain the systematic differences. PCSAs and fascicle lengths measured by D'Souza et al. (2019), who used similar MRI‐based methods as used in the present study, were on the high and low end of the distributions found in the present study, respectively. This difference may be explained by differences in ankle joint angles at which measurements were obtained, as D'Souza et al. (2019) obtained scans with the ankle in the resting angle, which was more plantarflexed than in the present study. This highlights the need for standardisation of joint positions at which measurements are obtained or ideally normalisation to optimal fascicle lengths by measuring sarcomere lengths. Data from other studies generally fell within the range reported here, suggesting broad applicability of the reference curves, at least for ages above 5 years. However, further research is needed to establish the validity of using these curves when measurements are obtained with ultrasound, which is more widely available in clinical settings than MRI.

Most previous studies on growth‐related changes in muscle architecture estimated linear effects of age on muscle architecture, but physical dimensions usually do not change linearly with age. Using quantile regression with a flexible, penalized b‐spline as a basis function on a large sample of children, we found that distributions of architectural parameters shift non‐linearly with age for most muscles and parameters. Quantile regression provides estimates, in the form of centile curves, of the distribution of values at any particular age. It is clear that the variance of these distributions was large for all parameters at all ages. Knowledge of the age‐ and sex‐conditional distribution of architectural parameters during typical childhood development may help identify impaired muscle growth on a subject‐ and muscle‐specific basis. As part of our current research, we are collecting data from children with cerebral palsy and intend to use the reference curves to identify impaired growth in muscles from children with cerebral palsy. We are also collecting longitudinal data from this cohort. The longitudinal data will be used to determine child‐specific growth trajectories, which cannot be inferred from the cross‐sectional data presented here. We expect the normative data presented here to be useful beyond this research program as well. For example, the normative data could be used to construct musculoskeletal models of children's legs or help clinicians plan muscle‐specific interventions in children with muscle growth disorders.

## AUTHOR CONTRIBUTIONS

BVYC, CM, CDR, DIW, IN, GCP, RDH, IKB and BB contributed to the study's conceptualisation and methodology. BVYC, SD, AL, RRNR, CYR, RDH and BB contributed to participant recruitment, and data collection and curation. BVYC, DIW, GCP, DIW, RDH and BB contributed to software development. BVYC, DIW, GCP, RDH and BB contributed to data analysis and interpretation. BVYC, RDH and BB contributed to the drafting of the original manuscript. All authors contributed to the revision and approval of the final manuscript.

## Supporting information


**Data S1:** supporting Information.

**Data S2:** supporting Information.

## Data Availability

The data will be available upon request from the corresponding author. Data can be shared for research purposes only.
