# Supplementary material for: Architecture of lower leg muscles in children: Reference curves and potential mechanisms of growth
Source: J Anat. 2025 Dec 1;249(1):54–67. doi: 10.1111/joa.70082 (PMC13238910; doi:10.1111/joa.70082)
Supplement: Supplementary file 1 — Data S1: supporting Information. [file JOA-249-54-s002.pdf]

Supplementary material 1

Reference curves for physiological cross-sectional area (PCSA) as a function of age. Each panel shows the 10th (bottom line), 50th (thick line in the middle) and 90th centile curves (top line) and the observations on muscles of individual boys (green, *n*=114, left panel) and girls (purple, *n*=83, right panel) on which the curves were fitted.

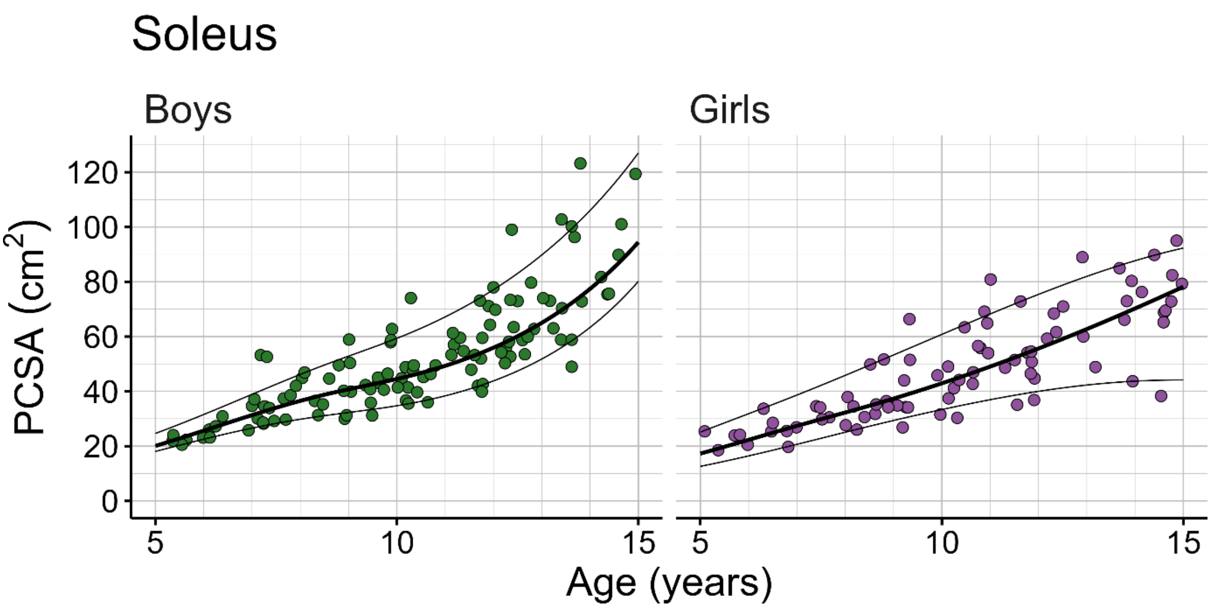

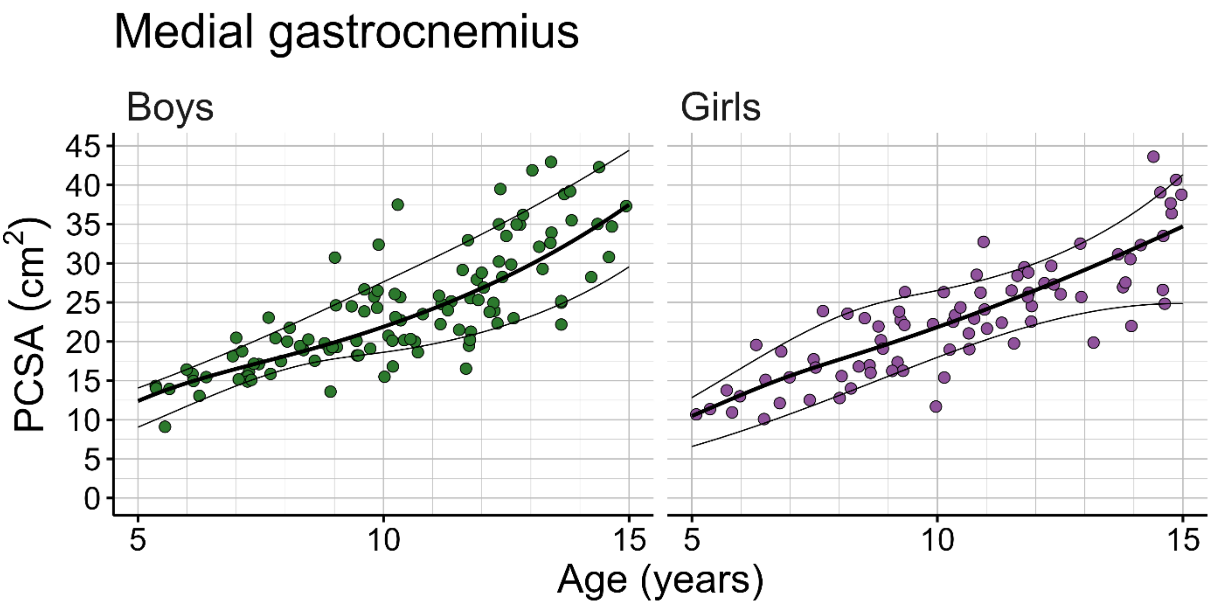

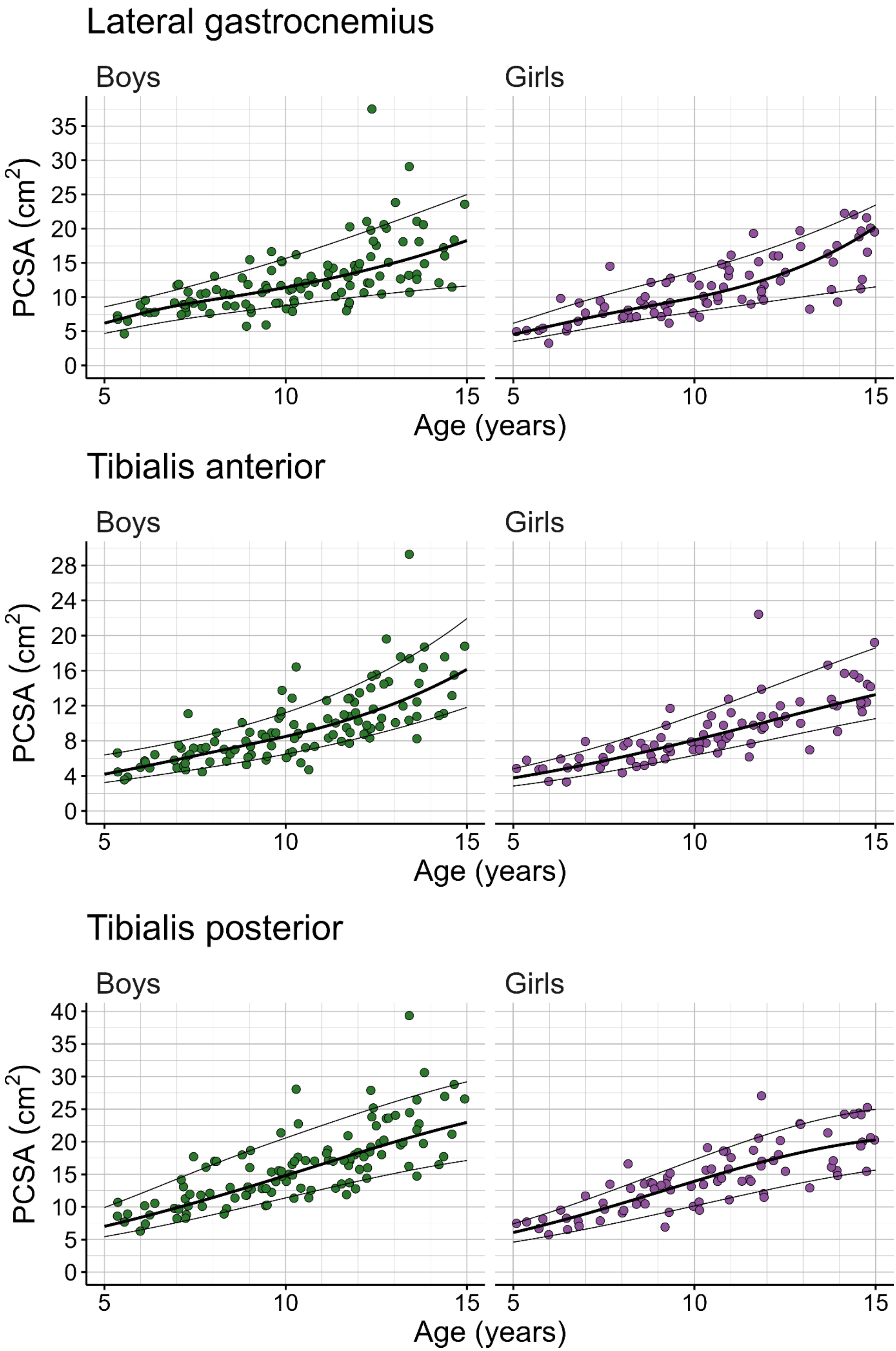

Lateral gastrocnemius
Boys
Girls
PCSA (cm^2)
Age (years)
Tibialis anterior
Boys
Girls
PCSA (cm^2)
Age (years)
Tibialis posterior
Boys
Girls
PCSA (cm^2)
Age (years)

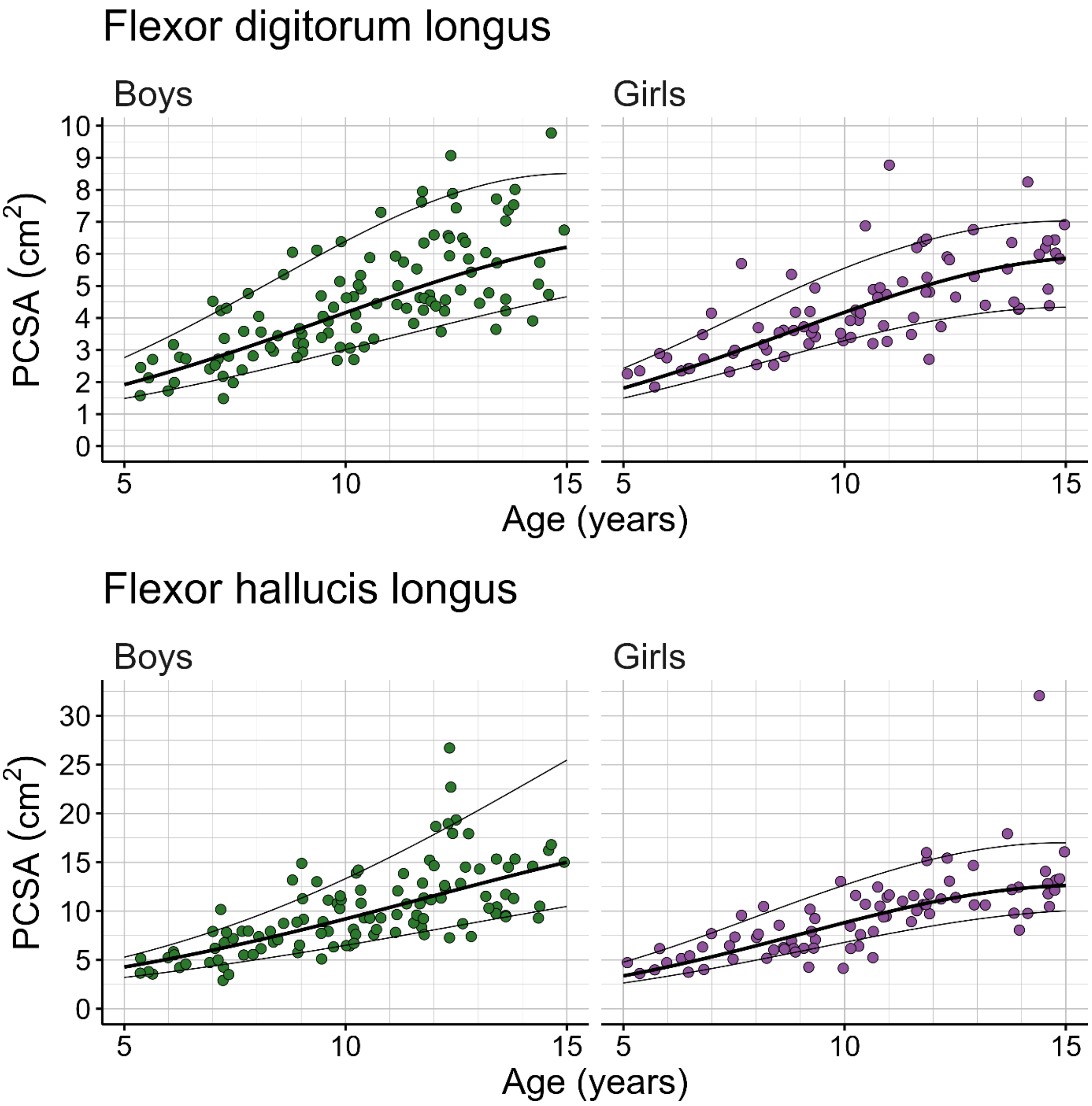

Flexor digitorum longus
Boys
Girls
PCSA (cm^2)
Age (years)
Flexor hallucis longus
Boys
Girls
PCSA (cm^2)
Age (years)

Reference curves for fascicle length as a function of age. Each panel shows the 10th (bottom line), 50th (thick line in the middle) and 90th centile curves (top line) and the observations on muscles of individual boys (green, *n*=114, left panel) and girls (purple, *n*=83, right panel) on which the curves were fitted.

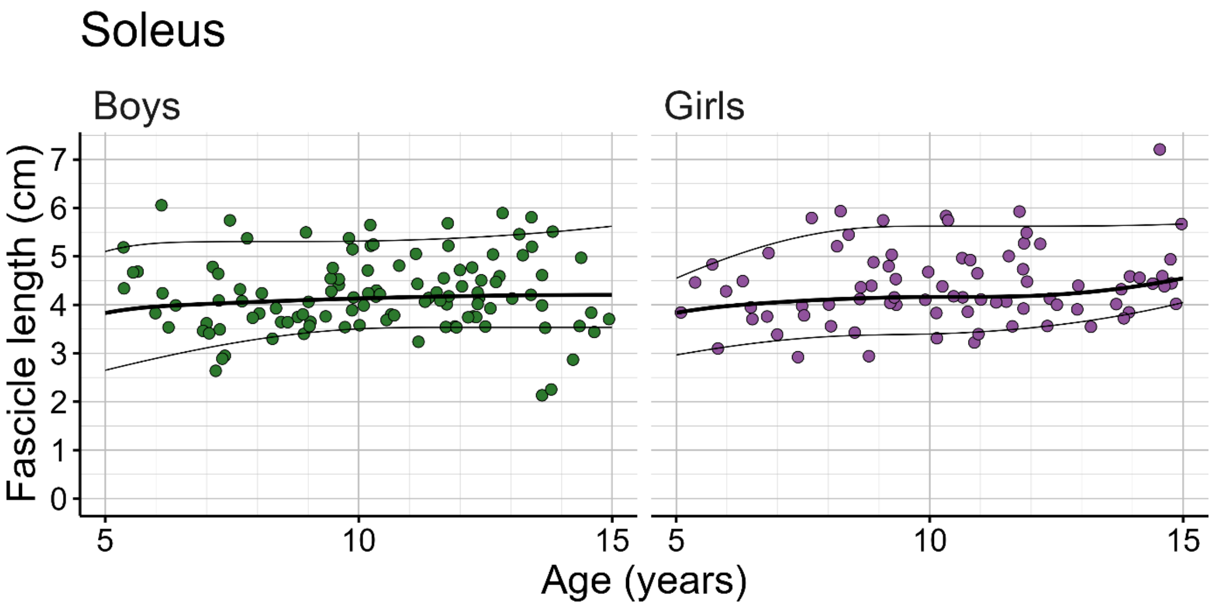

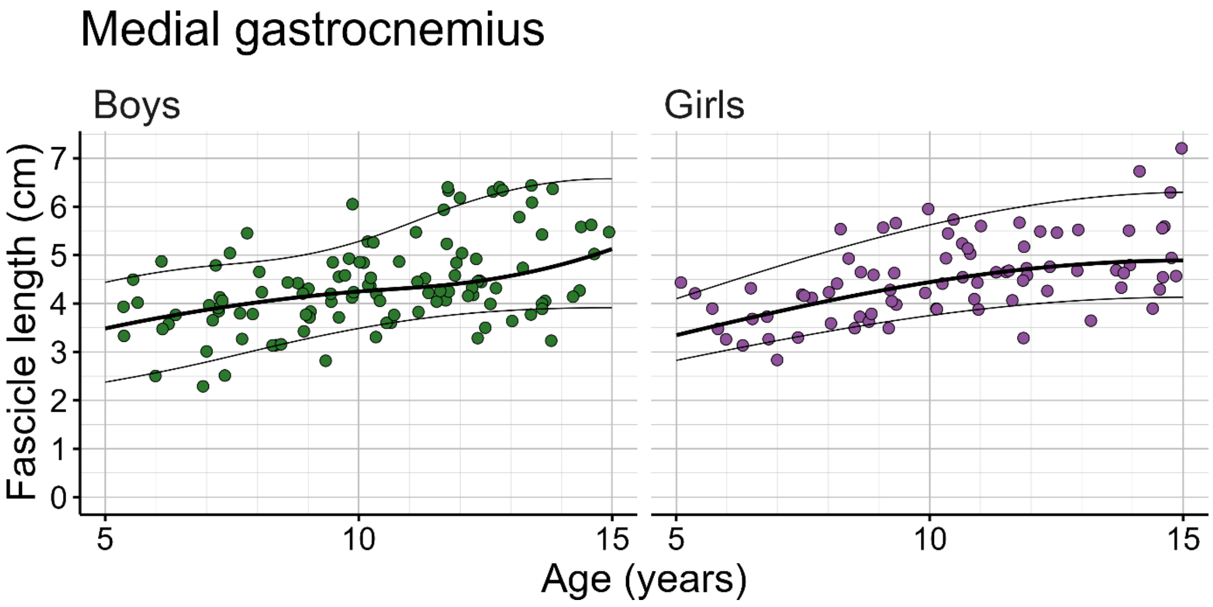

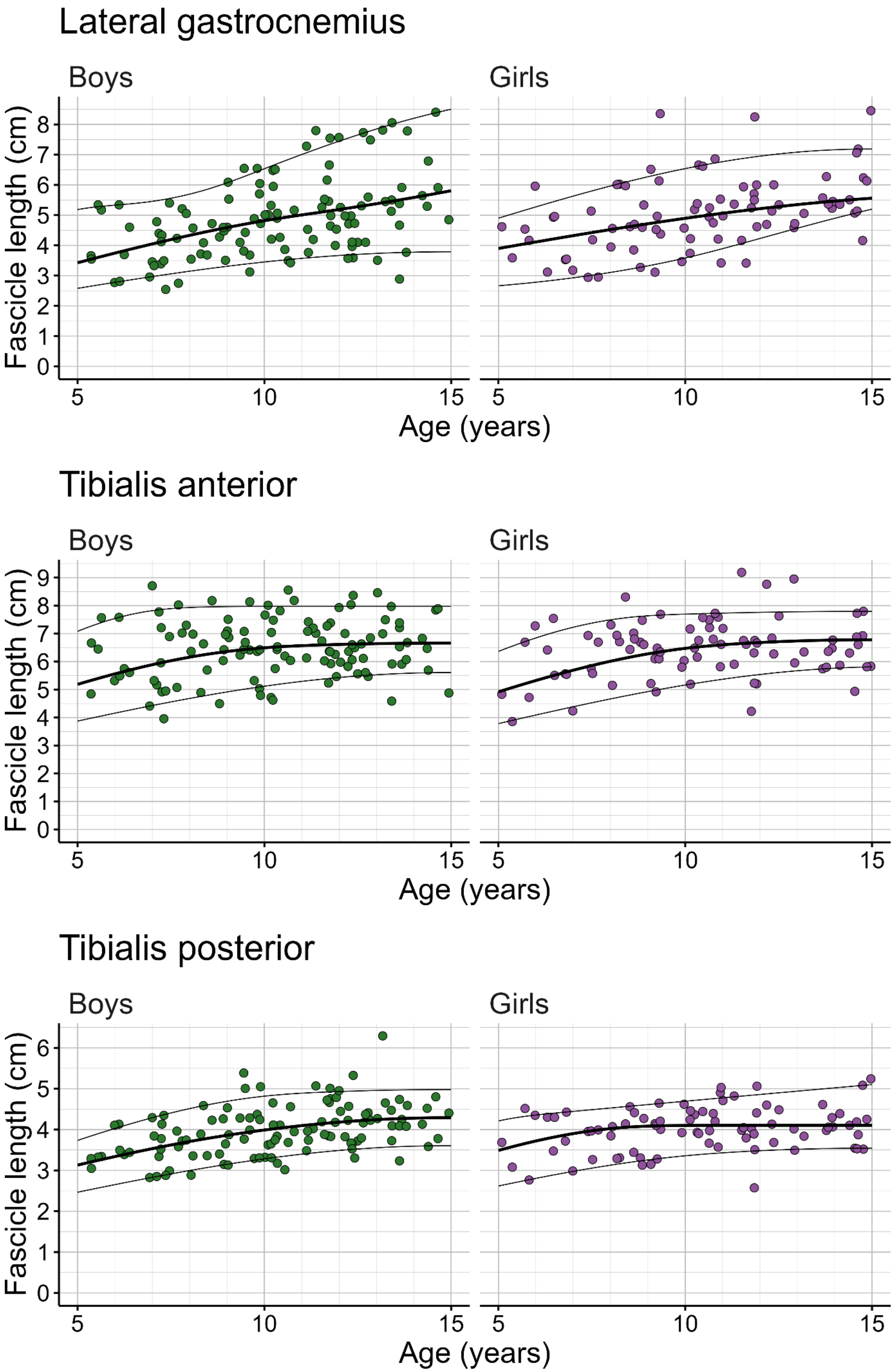
Lateral gastrocnemius
Boys
Girls
Fascicle length (cm)
Age (years)
Tibialis anterior
Boys
Girls
Fascicle length (cm)
Age (years)
Tibialis posterior
Boys
Girls
Fascicle length (cm)
Age (years)

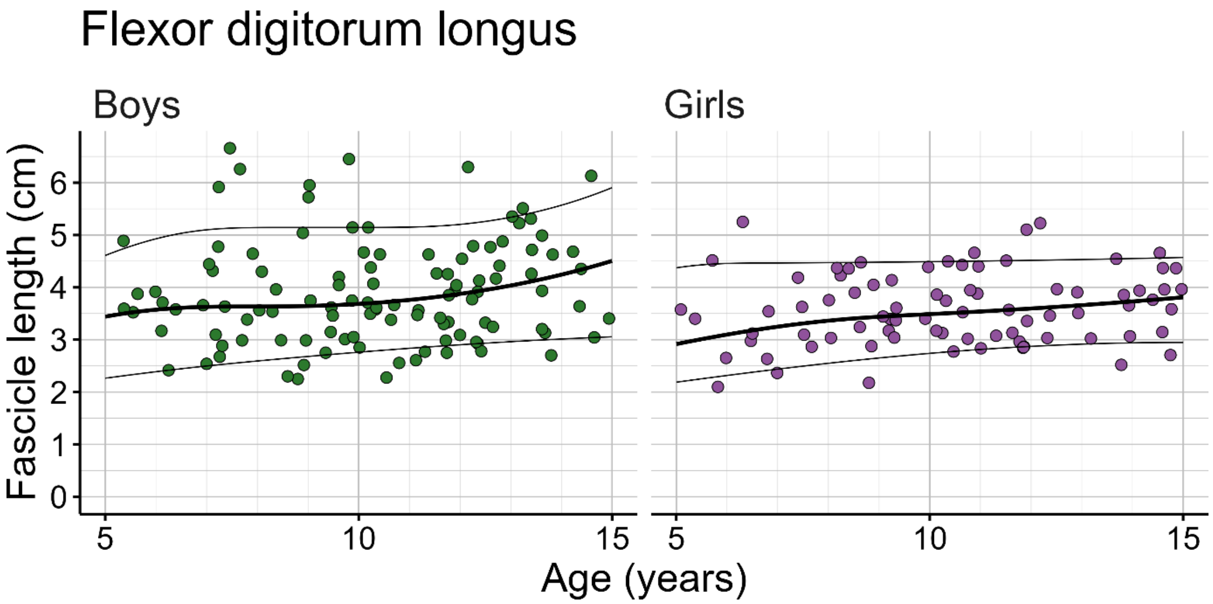

Flexor digitorum longus
Boys
Girls
Fascicle length (cm)
0
1
2
3
4
5
6
5
10
15
Age (years)

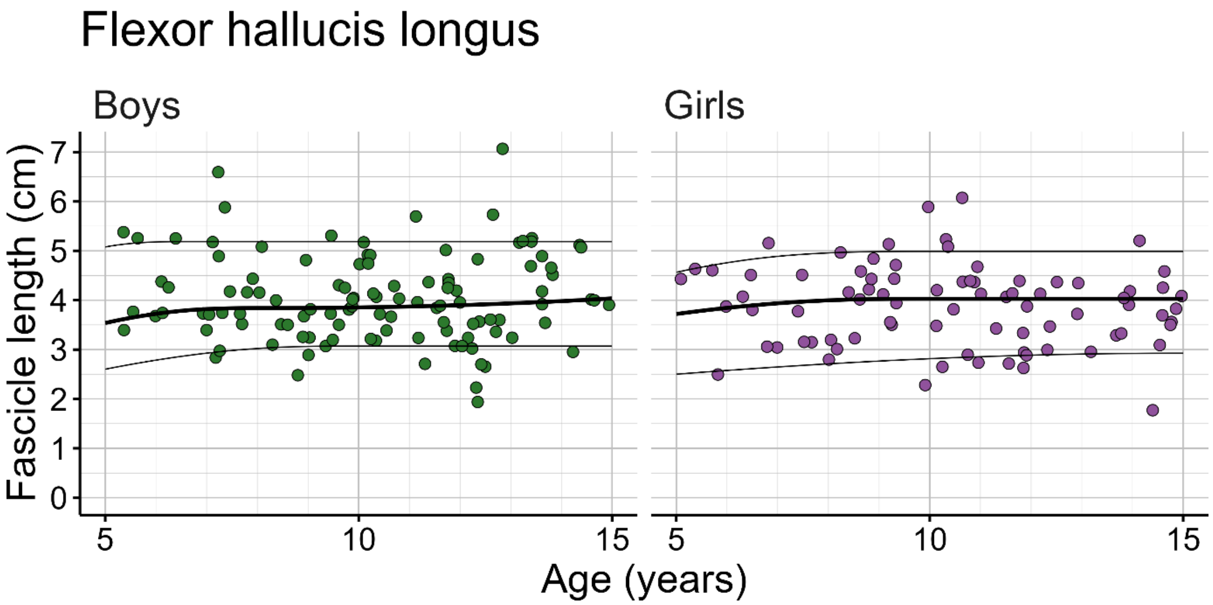

Flexor hallucis longus
Boys
Girls
Fascicle length (cm)
0
1
2
3
4
5
6
7
5
10
15
Age (years)

Reference curves for pennation angle as a function of age. Each panel shows the 10th (bottom line), 50th (thick line in the middle) and 90th centile curves (top line) and the observations on muscles of individual boys (green, *n*=114, left panel) and girls (purple, *n*=83, right panel) on which the curves were fitted.

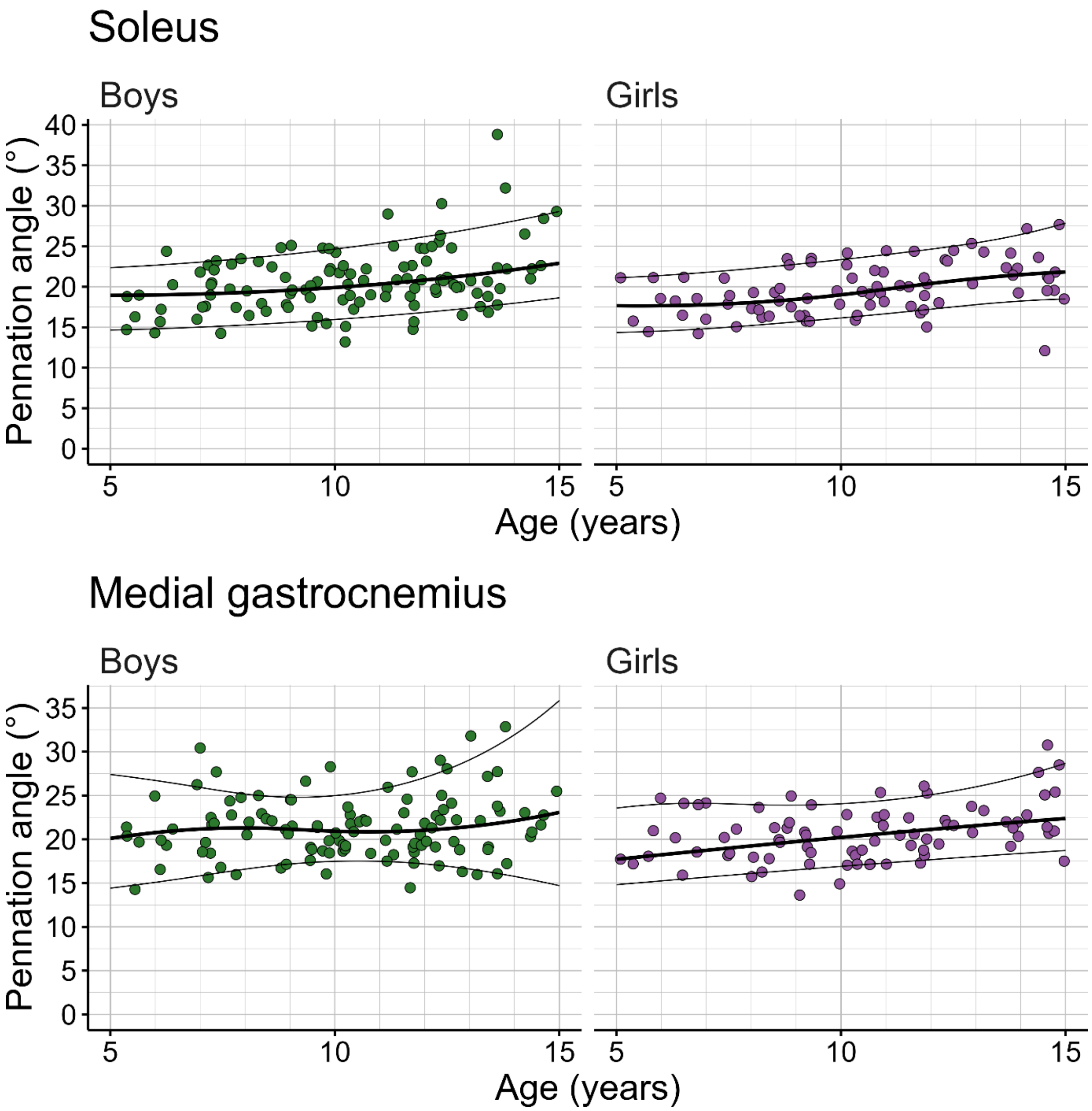

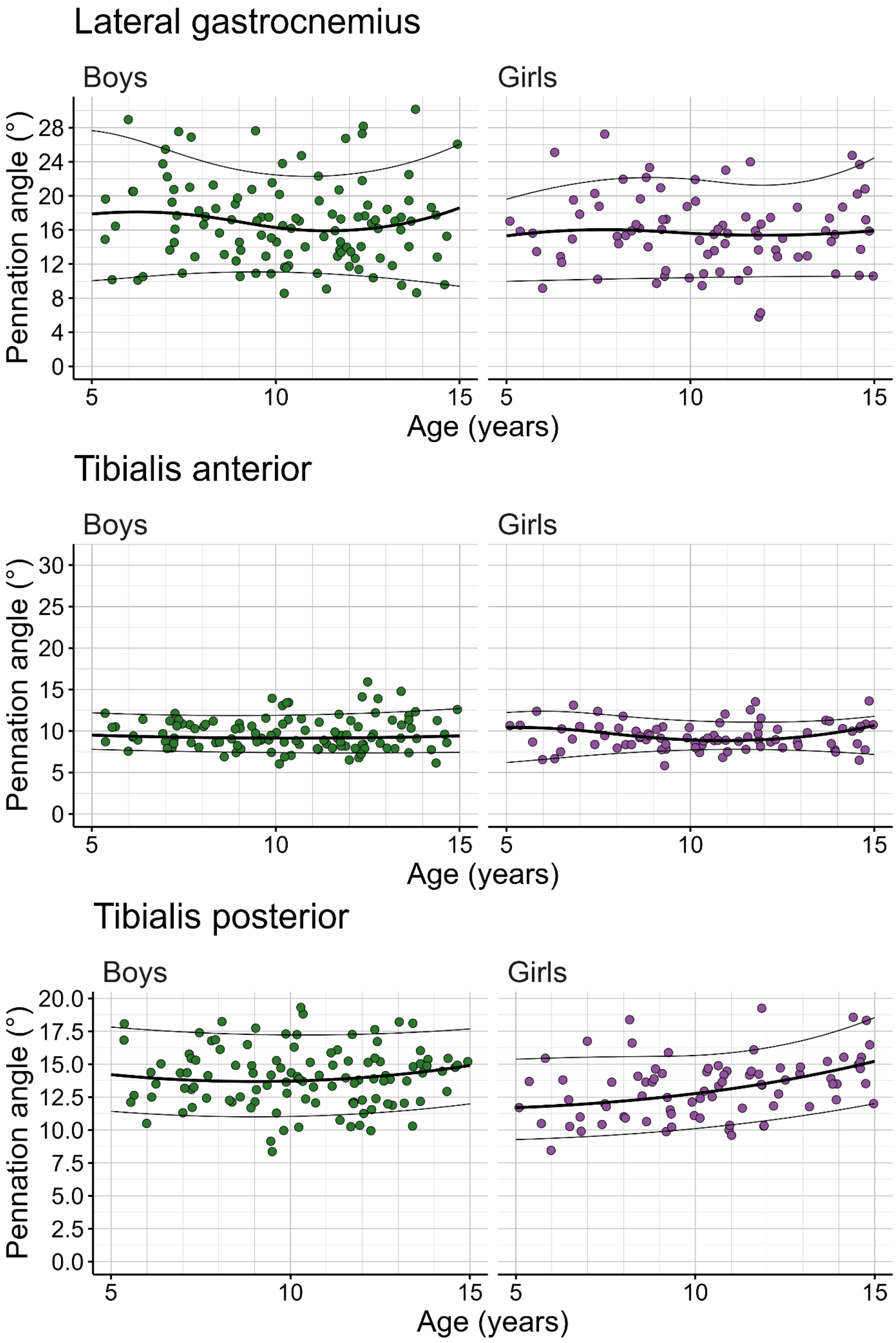
Lateral gastrocnemius
Boys
Girls
Pennation angle (°)
Age (years)
Tibialis anterior
Boys
Girls
Pennation angle (°)
Age (years)
Tibialis posterior
Boys
Girls
Pennation angle (°)
Age (years)

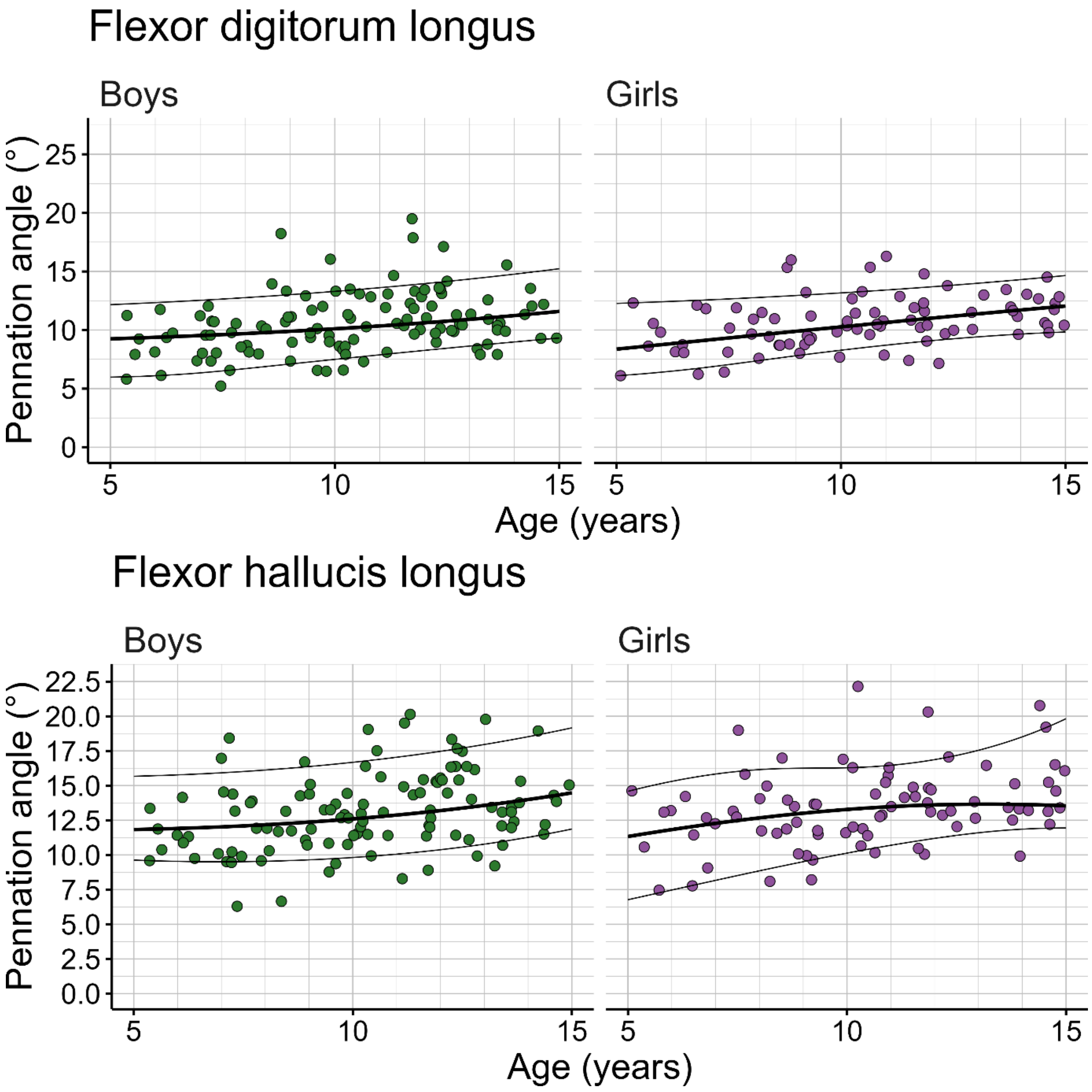
Flexor digitorum longus
Boys
Girls
Pennation angle (°)
25
20
15
10
5
0
5
10
15
Age (years)
Flexor hallucis longus
Boys
Girls
Pennation angle (°)
22.5
20.0
17.5
15.0
12.5
10.0
7.5
5.0
2.5
0.0
Age (years)
