# Supplementary material for: Architecture of lower leg muscles in children: Reference curves and potential mechanisms of growth
Source: J Anat. 2025 Dec 1;249(1):54–67. doi: 10.1111/joa.70082 (PMC13238910; doi:10.1111/joa.70082)
Supplement: Supplementary file 2 — Data S2: supporting Information. [file JOA-249-54-s001.pdf]

Supplementary Information 2

Table S1 Linear effect of age on fascicle, aponeurosis, tendon and muscle-tendon-unit length for seven lower leg muscles.

Variable	Muscle	Intercept	Effect of age (cm/yr) (95% confidence interval)	p-value
**Tibia length**	**-**	15.96	1.60 (1.50 to 1.71)	<0.001
**Fascicle length**	**Soleus**	3.90	0.01 (-0.03 to 0.06)	0.60
	**Medial gastrocnemius**	2.60	0.15 (0.11 to 0.19)	<0.001
	**Lateral gastrocnemius**	2.58	0.21 (0.15 to 0.28)	<0.001
	**Tibialis anterior**	5.67	0.07 (0.01 to 0.13)	<0.05
	**Tibialis posterior**	3.08	0.08 (0.05 to 0.11)	<0.001
	**Flexor digitorum longus**	3.36	0.03 (-0.02 to 0.08)	0.18
	**Flexor hallucis longus**	4.11	-0.02 (-0.07 to 0.02)	0.34
**Aponeurosis length**	**Soleus**	9.21	1.36 (1.24 to 1.48)	<0.001
	**Medial gastrocnemius**	9.36	0.70 (0.61 to 0.79)	<0.001
	**Lateral gastrocnemius**	8.12	0.53 (0.43 to 0.63)	<0.001
	**Tibialis anterior**	6.21	1.33 (1.22 to 1.44)	<0.001
	**Tibialis posterior**	9.73	1.21 (1.12 to 1.30)	<0.001
	**Flexor digitorum longus**	4.88	1.16 (1.05 to 1.26)	<0.001
	**Flexor hallucis longus**	5.69	0.70 (0.56 to 0.83)	<0.001
**Tendon length**	**Soleus**	0.59	0.20 (0.12 to 0.28)	<0.001
	**Medial gastrocnemius**	5.79	0.82 (0.71 to 0.92)	<0.001
	**Lateral gastrocnemius**	6.43	0.93 (0.83 to 1.04)	<0.001
	**Tibialis anterior**	1.39	0.08 (0.04 to 0.12)	<0.001
	**Tibialis posterior**	0.75	0.18 (0.15 to 0.20)	<0.001
	**Flexor digitorum longus**	0.37	0.10 (0.07 to 0.12)	<0.001
	**Flexor hallucis longus**	-0.20	0.04 (0.03 to 0.06)	<0.001
**Muscle-tendon-unit length**	**Soleus**	13.70	1.57 (1.47 to 1.67)	<0.001
	**Medial gastrocnemius**	17.74	1.67 (1.56 to 1.77)	<0.001
	**Lateral gastrocnemius**	17.13	1.68 (1.57 to 1.78)	<0.001
	**Tibialis anterior**	13.27	1.48 (1.37 to 1.59)	<0.001
	**Tibialis posterior**	13.56	1.46 (1.36 to 1.56)	<0.001
	**Flexor digitorum longus**	8.60	1.29 (1.18 to 1.40)	<0.001
	**Flexor hallucis longus**	9.61	0.72 (0.57 to 0.86)	<0.001
